# Identification and Expression Analysis of CCCH Zinc Finger Family Genes in *Oryza sativa*

**DOI:** 10.3390/genes16040429

**Published:** 2025-04-03

**Authors:** Zhihan Wang, Shunyuan Li, Hongkai Wu, Linzhou Huang, Liangbo Fu, Chengfang Zhan, Xueli Lu, Long Yang, Liping Dai, Dali Zeng

**Affiliations:** 1 College of Advanced Agricultural Sciences, Zhejiang A&F University, Hangzhou 311300, China; 2 State Key Laboratory for Rice Biology, China National Rice Research Institute, Chinese Academy of Agricultural Sciences, Hangzhou 311400, China

**Keywords:** CCCH gene family, rice, gene expression, haplotype analysis, phenotypic analysis

## Abstract

Background: CCCH zinc finger proteins (OsC3Hs) are a class of transcriptional regulators that play important roles in plant development and stress responses. Although their functional significance has been widely studied in model species, comprehensive genome-wide characterization of CCCH proteins in rice (*Oryza sativa*) remains limited. Methods: Using *Arabidopsis* CCCH proteins as references, we identified the CCCH gene family in rice and analyzed the physicochemical properties, subcellular localization, conserved structures, phylogeny, cis-regulatory elements, synteny analysis, spatiotemporal expression patterns, and expression patterns under drought, ABA, and MeJA treatments for the identified CCCH family members. Results: The results showed that the rice CCCH family comprises 73 members, which are unevenly distributed across the 12 chromosomes. Phylogenetic analysis classified them into 11 subfamilies. Subcellular localization indicated that most members are localized in the nucleus. The upstream regions of CCCH promoters contain a large number of cis-regulatory elements related to plant hormones and biotic stress responses. Most genes respond to drought, abscisic acid (ABA), and methyl jasmonate (MeJA) treatments. *OsC3H36* was highly expressed under drought, ABA, and MeJA treatments. Haplotype analysis of this gene revealed two major allelic variants (H1 and H2), with H1 predominantly found in japonica rice and associated with increased grain width and 1000-grain weight. Functional validation using a chromosome segment substitution line (CSSL1) confirmed these findings. Conclusions: CCCH genes play important roles in rice growth, development, and stress responses. Additionally, we validated that OsC3H36 is associated with rice grain width and 1000-grain weight.

## 1. Introduction

Zinc finger proteins (ZFPs), characterized by their zinc finger domains, represent one of the largest transcription factors (TF) family in plants. These proteins play crucial roles in regulating transcription and orchestrating various biological processes [1,2,3]. Zinc finger domains are primarily composed of cysteine and/or histidine residues, and, based on the number and arrangement of these amino acids, ZFPs can be categorized into nine major types: C2H2, C8, C6, C3HC4, C2HC, C2HC5, C4, C4HC3, and CCCH [4,5,6,7]. Members of the ZFP family are involved in critical cellular processes such as differentiation, proliferation, and apoptosis through the modulation of transcriptional and post-transcriptional regulation of target genes [6,8].

The CCCH zinc finger protein family is characterized by its signature CCCH-type motifs, each consisting of three cysteine residues and one histidine residue arranged in a conserved sequence pattern: C-X_4–15_-C-X_4–6_-C-X_3–4_-H. In this motif, “C” denotes cysteine, “H” represents histidine, and “X” corresponds to any intervening amino acids [9]. Based on the number and spatial distribution of these CCCH motifs, CCCH zinc finger proteins are further classified into two subgroups: tandem CCCH-type zinc finger (TZF) proteins and non-TZF proteins. Both TZF and non-TZF play vital roles in diverse biological processes, including development and responses to biotic and abiotic stresses [10]. In *Arabidopsis*, extensive research has revealed the functional significance of CCCH zinc finger proteins in various physiological and stress-related pathways. For example, HUA1 in *A. thaliana* is a TZF protein with six tandem CCCH motifs, which has been demonstrated to be an RNA-binding protein. As a nuclear transcriptional activator, it promotes seed germination, seedling development, and induces early flowering by binding to specific promoters [11,12,13]. Along with HUA2 and HEN4, HUA1 also facilitates floral morphogenesis by enhancing the processing of AGAMOUS pre-mRNA [12]. Additionally, the seed-specific AtTZF4 is implicated in pigment-mediated seed germination [14]. Another notable CCCH protein, AtCPSF30, exhibits RNA-binding activity and interacts with calmodulin. Its RNA-binding capacity is dynamically regulated by calmodulin and calcium ion concentrations [15].

CCCH-type zinc proteins have been extensively characterized as key regulators of abiotic stress responses across various plant species. In cotton, GhZFP1 enhances stress tolerance by interacting with GZIRD21A and GZIPR5, and its expression is upregulated under salt stress, osmotic stress, and salicylic acid treatment [16]. Similarly, in *A. thaliana*, HUA1 has been demonstrated to play a crucial role in stress adaptation by positively regulating ABA-dependent signaling pathways, thereby enhancing plant tolerance to osmotic, oxidative, and salt stresses [10]. Molecular genetic studies have identified additional *A. thaliana* CCCH proteins involved in stress responses, AtSZF1 and AtSZF2, which have been characterized as salt stress regulators, with overexpression of *AtSZF1* showing significantly enhanced salt tolerance [17]. AtTZF3 and AtTZF2 are involved in ABA and JA signaling pathways, modulating responses to drought stress and controlling transpiration rates [18]. AtTZF1 exhibits dual regulatory functions, acting as a positive regulator of ABA and sugar signaling while simultaneously serving as a negative regulator of gibberellin (GA) metabolism. This unique regulatory capacity enables AtTZF1 to enhance cold and drought tolerance when overexpressed. Furthermore, AtTZF4/5/6 have been implicated in the regulation of seed germination through their involvement in ABA- and GA-mediated signaling pathways [19].

As a staple cereal crop that sustains nearly half of the world’s population, rice exhibits significant vulnerability to various biotic and abiotic stresses, which substantially constrain its global productivity. In China, where rice serves as the principal dietary staple for more than 60% of the population, these challenges are particularly acute. Given the well-documented involvement of CCCH-type zinc finger proteins in regulating plant developmental processes and stress adaptation mechanisms, systematic characterization of their functional roles in rice represents a critical research priority. In this study, we conducted genome-wide identification and functional annotation of genes encoding 73 CCCH zinc finger proteins (designated as OsC3Hs) in rice. Our comprehensive analysis included phylogenetic relationships, protein domain architecture characterization, conserved motif identification, and cis-regulatory element prediction. Furthermore, we performed extensive expression profiling to determine the tissue-specific expression patterns and stress-responsive behaviors of these genes under various hormonal treatments and abiotic stress conditions, thereby providing valuable insights into their potential biological functions.

## 2. Materials and Methods

### 2.1. Identification of CCCH Proteins in Rice

The sequences of CCCH proteins in *A*. *thaliana* were retrieved from the Phytozome database (https://phytozome-next.jgi.doe.gov/, accessed on 17 December 2024) [20] and subsequently used as query sequences to identify homologous CCCH proteins in rice (*Oryza sativa*) via BLASTP searches in Phytozome, with an E-value cutoff of <1 × 10^−5^. To confirm the presence of CCCH domains in the identified proteins, additional domain analysis was conducted using multiple databases, including Pfam (PF00642, http://pfam.xfam.org/, accessed on 17 December 2024) [21], SMART (SM00356, http://smart.embl-heidelberg.de/, accessed on 17 December 2024) [22], InterPro (https://www.ebi.ac.uk/interpro/, accessed on 17 December 2024) [23], and NCBI Conserved Domain Database (CDD, https://www.ncbi.nlm.nih.gov/cdd, accessed on 17 December 2024) [24].

The rice CCCH proteins were subsequently renamed based on their chromosomal positions. The isoelectric points (pI), molecular weights (MW), and protein lengths of the OsC3H proteins were calculated using the ExPASy ProtParam tool (https://web.expasy.org/protparam/, accessed on 17 December 2024) [25]. Subcellular localization predictions for the OsC3H proteins were performed using WoLF PSORT (https://wolfpsort.hgc.jp/, accessed on 17 December 2024) [26].

### 2.2. Phylogeny and Protein Structure Analysis of the OsC3H Family Members

Using the Pfam (PF00642), SMART (SM00356), InterPro, and NCBI CDD databases, the location of the CCCH protein domain and other functional protein domains were confirmed. Additionally, the MEME website was used to predict 10 conserved motifs of OsC3H proteins [27]. Multiple sequence alignments of the OsC3H protein sequences were performed using the MUSCLE method implemented in MEGA11, with default parameters [28]. Based on these alignments, a phylogenetic tree was constructed using the neighbor-joining (NJ) method with 1000 bootstrap replicates to assess the reliability of the branches. The same approach was applied to construct phylogenetic trees for CCCH proteins in both rice and *A. thaliana*.

### 2.3. OsC3H Family Genomic Localization, Gene Duplication, and Synteny Analysis

The visualization of OsC3H family genes was performed using TBtools v2.154 [29]. Synteny relationships within the rice genome and between rice (*O. sativa*) and other plant species, including *Zea mays*, *Panicum virgatum*, *Triticum aestivum*, *Hordeum vulgare*, *A. thaliana*, *Glycine max*, *Brassica napus*, *Brassica rapa*, *Medicago truncatula*, *Solanum lycopersicum*, *Citrus clementina*, *Vitis vinifera*, and *Populus trichocarpa*, were analyzed using the MCScanX plugin in TBtools [29].

To estimate the evolutionary rates of the identified genes, the ratio of non-synonymous substitutions (Ka) to synonymous substitutions (Ks) was calculated using the KaKs_Calculator 3.0 [30]. The Ka/Ks ratio served as an indicator of the selection pressure acting on the genes.

### 2.4. Promoter Analysis of CCCH Genes in Rice

The 2 kb upstream sequences of the OsC3H genes were extracted from the rice (*O*. *sativa* L.) genome using TBtools. The extracted sequences were analyzed for cis-regulatory elements using the PlantCARE database [31].

### 2.5. Analysis of OsC3H Gene Expression

Using 14-day rice seedlings as material, RNA was extracted from the root and shoot tissues of rice under ABA, MeJA, and drought treatments at 3 h, 6 h, 12 h, and 24 h to calculate expression levels. The data were downloaded from the PPRD database (http://ipf.sustech.edu.cn/pub/zmrna/tutorials.php, accessed on 30 December 2024) [32]. Additionally, the expression levels of the OsC3H gene family in different rice tissues were also downloaded from this database. The downloaded gene expression levels were normalized using log10 (FPKM + 1).

### 2.6. Haplotype Analysis of OsC3Hs

We downloaded the SNP genotyping data for 533 different rice varieties from the Ricevarmap database (http://ricevarmap.ncpgr.cn/, accessed on 10 January 2025) for haplotype analysis. We selected SNPs in the regions of 2 kb upstream of the gene, the gene itself, and 1 kb downstream. Genotypes with identical bases at all sites were classified as the same haplotypes. We then screened for two major haplotypes for further analysis. Additionally, based on the gene haplotypes, we downloaded the corresponding expression data from the 158 rice leaf expression datasets previously constructed in our laboratory [33]. We also downloaded the grain length, grain width, and grain weight data for 533 rice samples from the Ricevarmap database [34]. One-way ANOVA were used to determine whether there were significant differences in expression levels and phenotypes among different haplotypes (*p* < 0.05).

Based on the substitution lines previously constructed in our laboratory using 9311 as the recipient parent and Nipponbare as the donor parent [33], we screened for materials that matched the *OsC3H36* haplotypes. We measured the grain length and width of 20 seeds and conducted five measurements of 1000-grain weight, followed by *t*-tests (*p* < 0.05).

## 3. Results

### 3.1. Identification of CCCH Zinc Finger Proteins in Rice

Through genome-wide identification and systematic analysis, we characterized 73 CCCH genes in the rice genome. These genes were systematically renamed from *OsC3H1* to *OsC3H73* according to their chromosomal locations, following the sequential order from chromosomes 1 to 12 (Table 1). Bioinformatics characterization of the encoded OsC3H proteins revealed considerable heterogeneity in physicochemical properties. Protein lengths ranged from 146 to 1890 amino acid residues (mean = 535.9), predicted isoelectric points (pI) varied between 4.68 and 9.61, and molecular weights exhibited a broad range from 15.8 kDa to 207.2 kDa. Subcellular localization predictions indicated nuclear enrichment for the majority of CCCH proteins, with a minority localized to other cellular compartments (Table 1). Comprehensive molecular features of the rice CCCH zinc finger family are cataloged in Supplementary Table S1. Subcellular localization predictions indicated that most OsC3H proteins were likely localized in the nucleus (Table 1). Comprehensive details of the rice CCCH zinc finger family are provided in Table S1.

We further conducted a survey of CCCH genes and motifs across 14 species, including rice, *A. thaliana* [35], *Z. mays* [36], *H. vulgare* [37], *G. max* [38], *P. virgatum* [39], *B. napus* [40], *B. rapa* [41], *S. lycopersicum* [42], *M. truncatula* [42], *T. aestivum* [5], *V. vinifers* [43], *C. clementine* [44], and *P. trichocarpe* [45].

**Figure 1:**
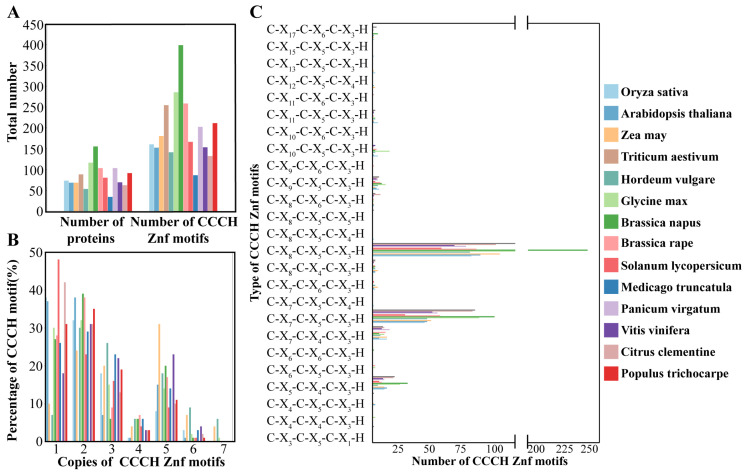
Characteristics and comparison of CCCH-type zinc finger proteins. (**A**) The number of CCCH proteins and CCCH motifs in *O*. *sativa*, *A*. *thaliana*, *Z*. *may*, *H*. *vulgare*, *G*. *max*, *P*. *virgatum*, *B*. *napus*, *B*. *rape*, *S*. *lycopersicum*, *M*. *truncatula*, *T*. *aestivum*, *V*. *vinifers*, *C*. *clementine*, and *P*. *trichocarpe*. (**B**) The proportion of CCCH proteins containing 1, 2, 3, 4, 5, 6, or 7 CCCH motifs in *O*. *sativa*, *A*. *thaliana*, *Z*. *may*, *H*. *vulgare*, *G*. *max*, *P*. *virgatum*, *B*. *napus*, *B*. *rape*, *S*. *lycopersicum*, *M*. *truncatula*, *T*. *aestivum*, *V*. *vinifers*, *C*. *clementine*, and *P*. *trichocarpe*. (**C**) The number of each type of CCCH motif in *O*. *sativa*, *A*. *thaliana*, *Z*. *may*, *H*. *vulgare*, *G*. *max*, *P*. *virgatum*, *B*. *napus*, *B*. *rape*, *S*. *lycopersicum*, *M*. *truncatula*, *T*. *aestivum*, *V*. *vinifers*, *C*. *clementine*, and *P*. *trichocarpe*.

While the numbers and types of CCCH motifs were reported in most species, information for *P. virgatum* and *T. aestivum* lacked motif-type details (Figure 1). The CCCH genes numbers ranged from 34 to 155 across these species, with corresponding motif counts varying between 86 and 398. In rice, 160 CCCH motifs were identified across 73 genes, placing it at an intermediate level among the species analyzed.

Each CCCH protein typically contained between one and six CCCH motifs. In rice, 27 OsC3H proteins were found to contain 1 CCCH motif, 24 proteins had 2 motifs, 13 had 3 motifs, 1 had 4 motifs, 6 had 5 motifs, and 2 had 6 motifs. This distribution aligns with trends observed in the other 13 species, where the majority of CCCH proteins possessed one or two motifs, followed by three or five motifs. Across the 14 species, a total of 25 distinct types of CCCH motifs were identified. Among these, two motifs—C-X_8_-C-X_5_-C-X_3_-H and C-X_7_-C-X_5_-C-X_3_-H—were the most prevalent and appeared to be ancestral forms of other CCCH motifs. Additionally, rare motif types, such as C-X_6_-C-X_5_-C-X_3_-H, were identified exclusively in specific species like tomato and soybean. Notably, rice contained a unique motif, C-X_8_-C-X_5_-C-X_4_-H, which was not found in any of the other species examined.

### 3.2. Phylogenetic Relationships, Protein Structures, and Conserved Domains of OsC3H Genes

**Figure 2:**
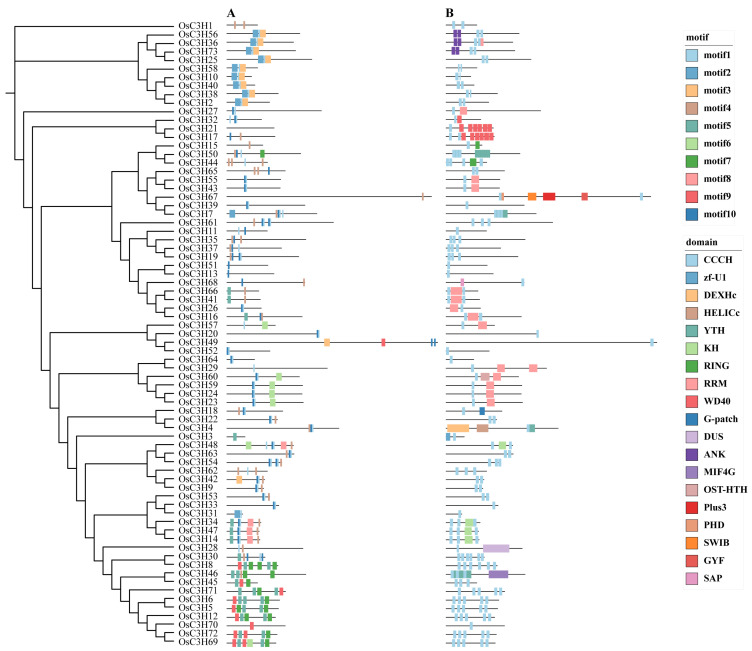
Phylogenetic relationships, motif composition, and domain analysis of rice CCCH genes. (**A**) Motifs of OsC3H proteins predicted by MEME, with different colors representing different motifs. (**B**) Domains of OsC3H proteins, with different colors indicating different domains.

To investigate the evolutionary relationships and structural characteristics of CCCH proteins, we performed a comprehensive phylogenetic and structural analysis using protein sequences from *A. thaliana* and rice. Multiple sequence alignment was conducted, followed by phylogenetic tree construction, revealing that rice CCCH proteins could be classified into 11 distinct subgroups (designated I-XI) based on the established classification criteria for *A. thaliana* CCCH proteins (Figure S1). The number of genes within each subgroup exhibited significant variation. To further investigate the evolutionary relationships within the rice CCCH gene family, we constructed a phylogenetic tree using MEGA11 software based on 73 OsC3H protein sequences. Additionally, motif and domain analyses were performed using MEME and multiple functional annotation databases. The results revealed that members within the same subgroup exhibited highly conserved motif compositions and domain distributions (Figure 2). Notably, in addition to the canonical CCCH domain, we identified multiple functional domains in these proteins. For example, subgroup VIII members OsC3H16, OsC3H41, and OsC3H66 were found to contain RNA recognition motif (RRM) domains. Furthermore, other subgroups contained proteins with additional functional domains, including WD40, ANK, and KH domains. These findings suggest that the CCCH domain may function cooperatively with other domains, potentially expanding the functional diversity and regulatory complexity of CCCH proteins in various biological processes.

### 3.3. Chromosomal Distribution and Gene Duplication of OsC3H Genes

**Figure 3:**
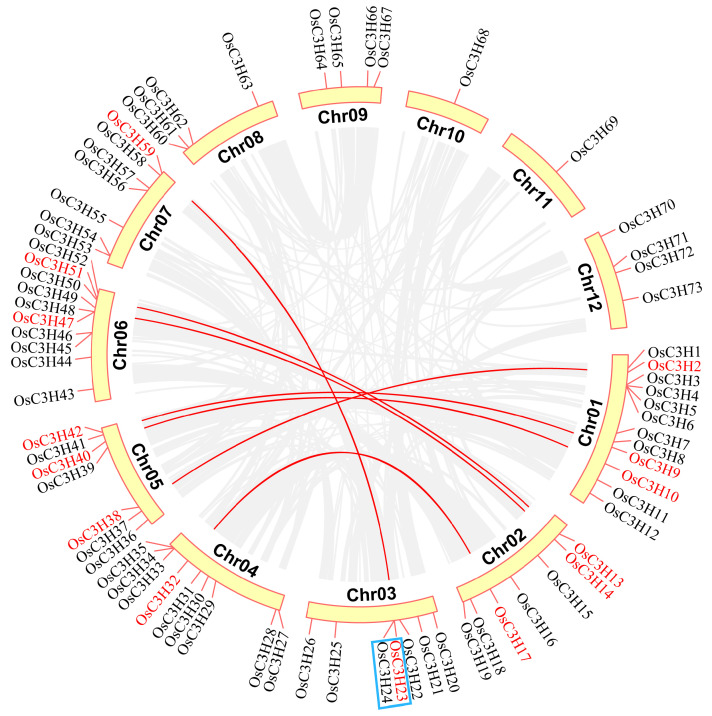
Distribution of CCCH genes on chromosomes and synteny relationships in rice. Red genes represent segmental duplicated genes, while blue boxes indicate tandem duplicated genes.

**Figure 4:**
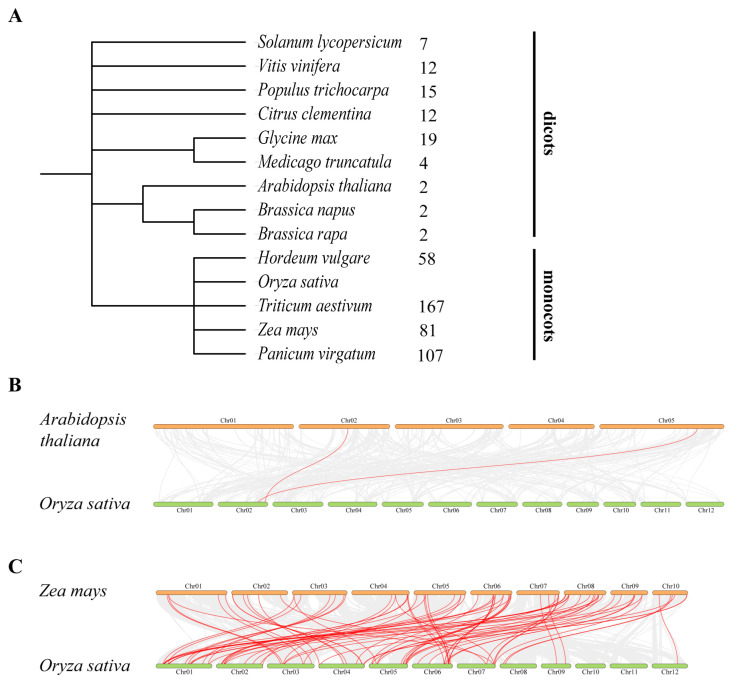
Phylogenetic relationships and synteny analysis of 14 Species. (**A**) Phylogenetic relationships among *O*. *sativa*, *A*. *thaliana*, *Zea may*, *H*. *vulgare*, *G*. *max*, *S. lycopersicum*, *P*. *virgatum*, *T*. *aestivum*, *B*. *napus*, *B*. *rape*, *M*. *truncatula*, *C*. *clementine*, *V*. *vinifera*, and *P*. *trichocarpe*, along with the number of homologous genes shared with rice from the other 13 species. (**B**) Synteny analysis between *O*. *sativa* and *A*. *thaliana*. The gray lines indicate collinear blocks between *O*. *sativa* and *A*. *thaliana*, and the red lines colinear gene pairs between *O*. *sativa* and *A*. *thaliana*. (**C**) Synteny analysis between *O*. *sativa* and *Z*. *may*. The gray lines indicate collinear blocks between *O*. *sativa* and *Z*. *may*, and the red lines colinear gene pairs between *O*. *sativa* and *Z*. *may*.

Chromosomal localization analysis of OsC3H genes revealed a markedly uneven distribution across the rice genome. Chromosome 1 harbored the highest number of OsC3H genes (12 genes), followed by chromosome 6 with 10 genes. In contrast, chromosomes 10 and 11 contained only one OsC3H gene each, representing the lowest distribution density (Figure 3). Gene duplication, a major driving force for gene family expansion in plants, plays a pivotal role in functional innovation and adaptive evolution. In this study, we identified eight duplicated gene pairs within the rice CCCH gene family, comprising one tandem duplication pair (*OsC3H23*/*OsC3H24*) and seven segmental duplication pairs. Phylogenetic analysis demonstrated that these duplicated gene pairs clustered within the same subfamily or branch. For instance, *OsC3H2*/*OsC3H38* were classified into subfamily XI. To assess the selective pressure acting on these duplicated OsC3H gene pairs, we calculated their Ka/Ks ratios. All eight duplicated gene pairs exhibited Ka/Ks values less than 1 (Table S2), indicating the action of purifying selection and suggesting limited functional divergence following duplication events. Furthermore, we investigated the syntenic relationships between rice and 13 other species, including 9 monocots and 4 dicots (Figure 4 and Supplementary Figures S2 and S3). The analysis revealed that the number of orthologous gene pairs between rice and the nine dicot species ranged from 2 to 19 (specifically, 7, 12, 15, 12, 19, 4, 2, 2, and 2), while the number of orthologous gene pairs between rice and the four monocot species ranged from 58 to 167 (specifically, 58, 167, 81, and 107) (Figure 4). The number of collinear gene pairs identified between rice and the dicots was 7, 12, 15, 12, 19, 4, 2, 2, and 2. In comparison, the number of collinear gene pairs between rice and the monocots was significantly higher, with 58, 167, 81, and 107 pairs identified, respectively (Figure 4). This discrepancy highlights a lower level of collinearity between rice and dicots compared with monocots, a finding that aligns with the phylogenetic relationships among these species.

### 3.4. Cis-Regulatory Elements Analysis in OsC3H Genes

**Figure 5:**
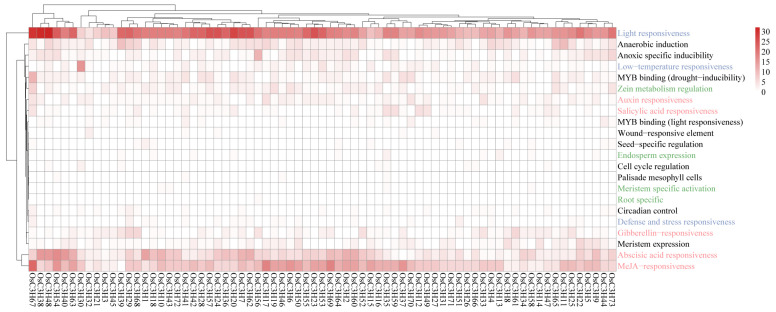
Number of cis-regulatory elements in rice OsC3Hs. Different font colors represent various types of elements. Blue, green, and red indicate stress response, tissue-specific expression, and plant growth regulators, respectively.

We analyzed the cis-regulatory elements in the promoter regions (2000 bp upstream of the start codon) of the OsC3H genes, identifying a total of 22 functional cis-regulatory elements (Figure 5). Among these, light-responsive elements were the most abundant, comprising the majority of the predicted elements (Figure 5, Table S3). Additionally, 11 types of hormone-responsive elements were identified, including auxin-responsive elements (TGA-element, AuxRR-core, AuxRE, and TGA-box), gibberellin-responsive elements (TATC-box, GARE-motif, and P-box), salicylic acid-responsive elements (TCA-element), and MeJA-responsive elements (CGTCA-motif and TGACG-motif). Notably, abscisic acid (ABA) and MeJA response elements were the most predominant, with 432 and 644 occurrences, respectively. Various biotic and abiotic stress-related regulatory elements were also observed in the promoter regions of OsC3H genes. A total of 339 anaerobic induction elements were detected across 66 OsC3H genes, including 205 ARE and 134 GC-motifs. Additionally, 85 low-temperature response elements (LTRs) were identified in 47 genes, and 128 drought-responsive elements were found in 58 genes. We also identified six types of cis-regulatory elements associated with plant organogenesis. These included meristem expression-related CAT boxes (53 genes), maize alcohol-soluble protein metabolism regulatory O2 sites (53 genes), and endosperm expression-related GCN4 motifs (10 genes). These results suggest that OsC3Hs play critical roles in regulating rice growth and development, mediating hormone signal transduction, and responding to various biotic and abiotic stresses.

### 3.5. Temporal–Spatial and Stress-Induced Expression Pattern Analysis of OsC3Hs

**Figure 6:**
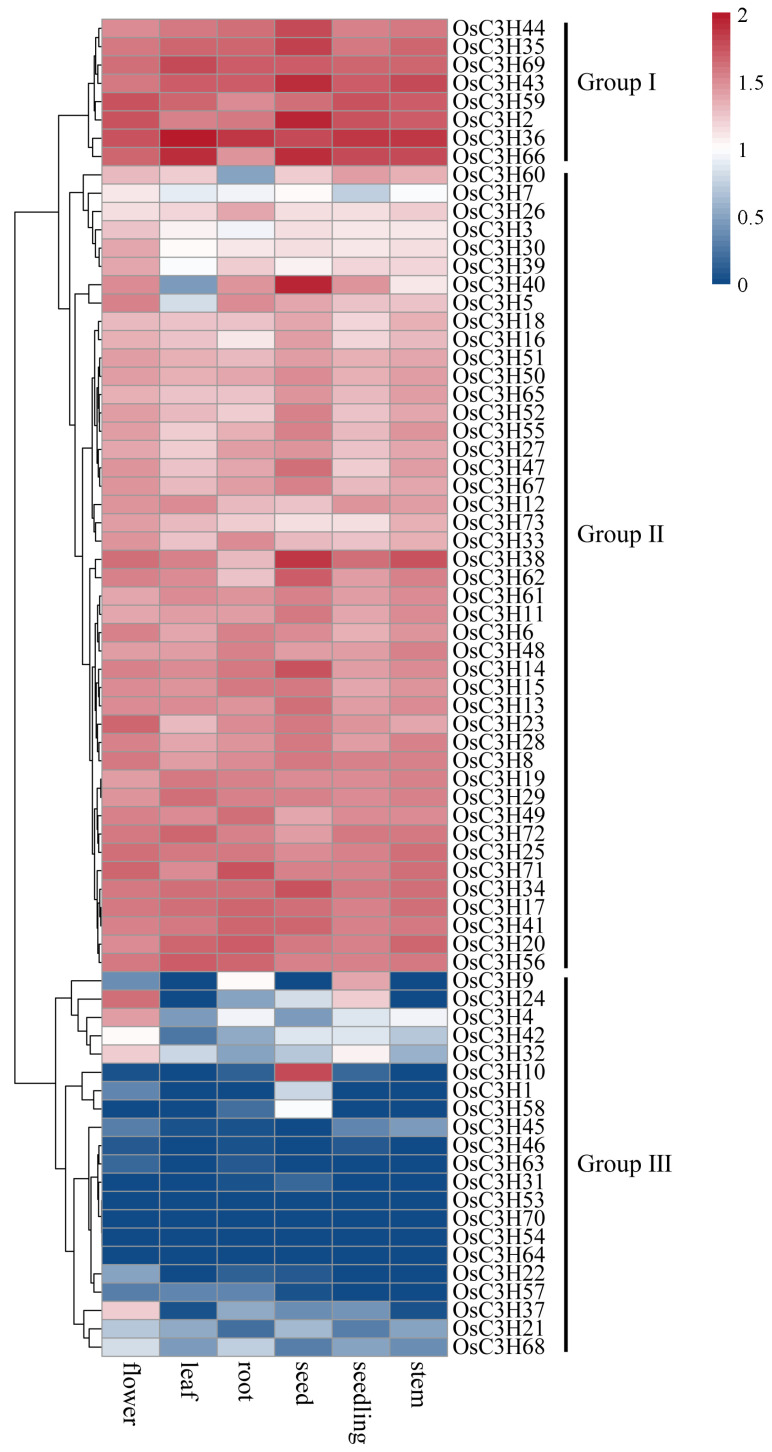
Expression profiles of OsC3Hs in different tissues.

**Figure 7:**
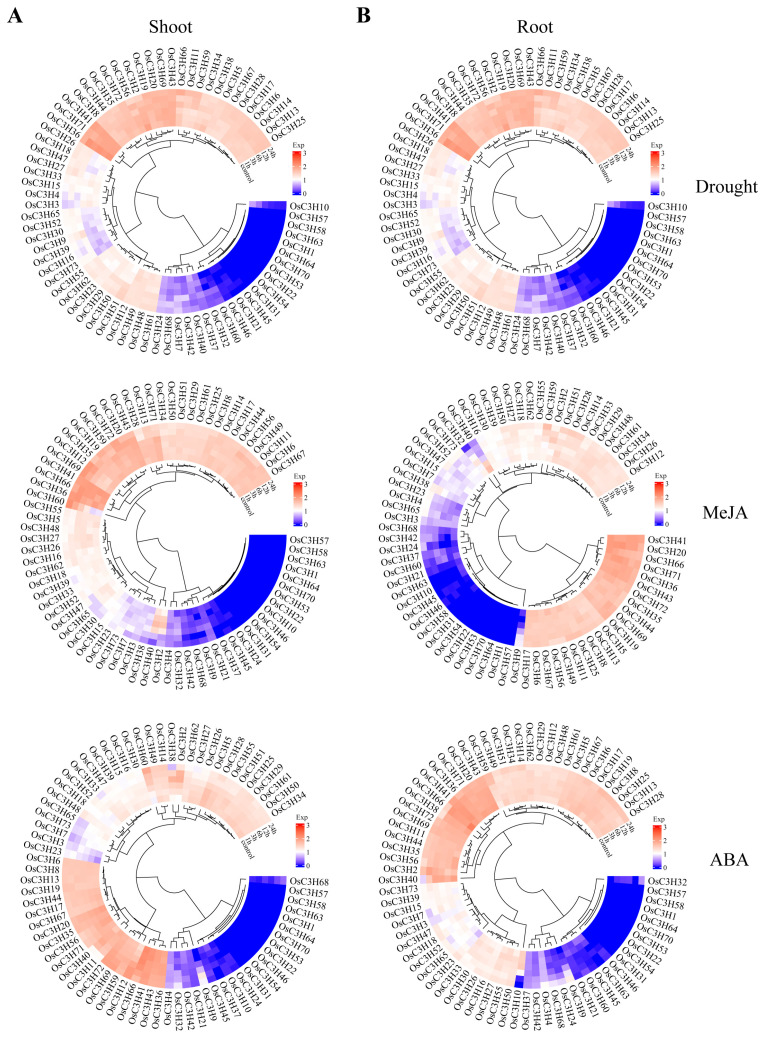
Expression levels in shoot and root of rice after drought, MeJA, and ABA treatments. (**A**) Expression levels in shoot. (**B**) Expression levels in root.

The analysis of tissue-specific expression profiles provided critical insights into the potential functions of genes in plants. Based on the expression data from six rice tissues, distinct expression patterns of OsC3H genes were observed (Figure 6). Genes in Group I exhibited high expression levels across all six tissues, whereas Group II genes showed moderate expression levels. In contrast, most genes in Group III displayed low or no expression in these tissues, although some exhibited notable tissue-specific expression. For instance, *OsC3H10* was predominantly expressed in seeds, while *OsC3H37* was specifically expressed in flowers. These findings suggest that different OsC3H genes may contribute to the development and function of specific rice tissues. In addition to tissue-specific expression, we analyzed the responses of OsC3H genes to drought stress and hormonal treatments with abscisic acid (ABA) and methyl jasmonate (MeJA). Expression levels were measured at six time points (0 h, 1 h, 3 h, 6 h, 12 h, and 24 h) in both shoot and root tissues (Figure 7). Under drought conditions, after 24 h, the expression of *OsC3H5*, *OsC3H38*, and *OsC3H40* in the shoot increased by 10.71-, 9.05-, and 3.47-fold, respectively. In the root, *OsC3H10* exhibited a remarkable 28.52-fold increase, despite being almost undetectable in the shoot. Similarly, during MeJA treatment, *OsC3H40* expression increased significantly, by 22.12-fold in the shoot and 21.96-fold in the root, after 24 h. Additionally, *OsC3H32* in the root exhibited a dramatic 45.98-fold increase. Following 24 h of ABA treatment, *OsC3H40* expression increased by 3-fold in the shoot and 16-fold in the root. These results demonstrate that certain OsC3H genes are highly responsive to drought, ABA, and MeJA stress, with differential expression patterns between tissues. This highlights their potential roles in mediating stress responses and contributing to tissue-specific adaptive mechanisms in rice.

### 3.6. Analysis of the CCCH Haplotype

**Figure 8:**
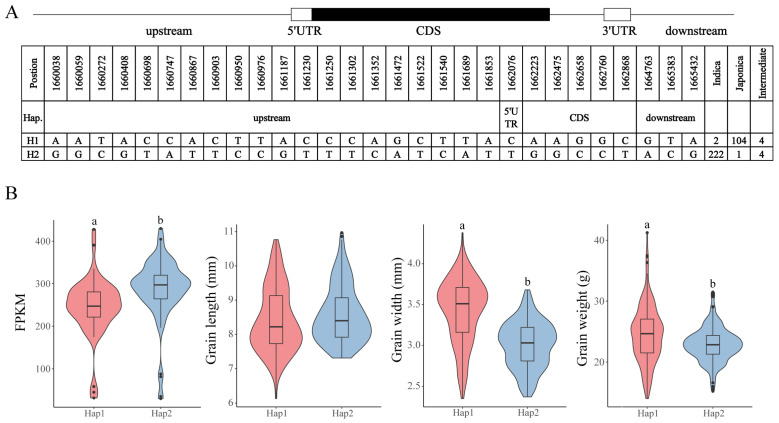
*OsC3H36* haplotype analysis. (**A**) The gene structure of *OsC3H36*. The coding region is indicated by a black box; 3′ UTR and 5′ UTR are indicated by white boxes. (**B**) Haplotype analysis of expression level and three phenotypes (grain length, grain width, grain weight). Different low-case letters above columns indicate statistical differences at *p* < 0.05. Red represents haplotype H1, and blue represents haplotype H2.

We selected *OsC3H36* for haplotype analysis based on its high expression levels across multiple tissues. *OsC3H36* exhibited two major haplotypes (H1 and H2), identified from 337 rice accessions, characterized by a total of 29 SNPs. Among these SNPs, 20 were located in the upstream regulatory region, 1 in the 5′ untranslated region (UTR), 5 in the coding sequence (CDS), and 3 in the downstream region (Figure 8A). Notably, the H1 haplotype was predominantly found in japonica rice varieties, whereas the H2 haplotype was primarily associated with indica rice varieties (Figure 8A, Table S4). To investigate the potential differential expression of *OsC3H36* between H1 and H2 haplotypes, we analyzed transcript abundance using RNA-seq data generated from leaf tissues available in our laboratory’s sequencing database [33]. The results revealed a statistically significant difference in the average gene expression levels between H1 and H2 haplotypes. Furthermore, phenotypic analysis indicated no significant difference in grain length between the two haplotypes; however, significant differences were observed in grain width and 1000-grain weight, suggesting a potential functional role of *OsC3H36* in regulating these agronomic traits (Figure 8B).

**Figure 9:**
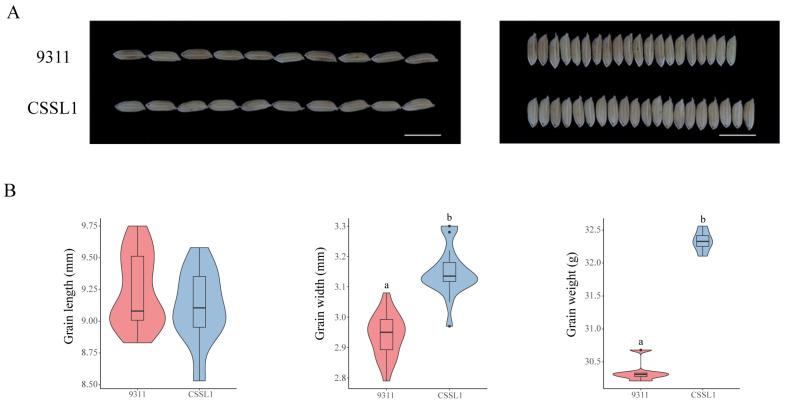
Phenotypes of 9311 and substitution lines. (**A**) Grain length (left) and grain width (right) of 9311 and substitution lines. (**B**) Grain length, grain width, and grain weight of 9311 and substitution lines. Different low-case letters above columns indicate statistical differences at *p* < 0.05. Red represents haplotype H1, and blue represents haplotype H2.

To validate whether the two allelic variants of *OsC3H36* exhibited significant differences in regulating grain weight, we screened one chromosome segment substitution line (CSSL1) from a CSSL library, with 9311 as the recurrent parent and Nipponbare (NPB) as the donor parent. Specifically, CSSL1 belonged to H1, while 9311 belonged to H2. We compared grain length, grain width, and 1000-grain weight between 9311 and CSSL1 (Figure 9). The results revealed no significant difference in grain length, but the grain width and 1000-grain weight of CSSL1 were significantly higher than those of 9311, indicating that the H1 haplotype was functionally superior in regulating grain width and weight. These findings were consistent with phenotypic data from the Ricevarmap database, further confirming the accuracy of our results.

## 4. Discussion

In this study, we conducted a comprehensive genome-wide identification and characterization of CCCH zinc finger proteins (OsC3Hs) in rice (*O*. *sativa* L.), identifying 73 members (Figure 2) and classifying them into 11 subgroups based on phylogenetic relationships (Figure S1). Structural and motif analyses revealed conserved domain architectures and the presence of additional functional motifs such as RNA recognition motifs (RRM) and WD40 domains, suggesting diverse functional roles (Figure 2B). Chromosomal distribution analysis highlighted an uneven genomic dispersion of OsC3H genes, with gene duplication events, particularly segmental duplications, playing a major role in their evolutionary expansion (Figure 3). Cis-regulatory element analysis revealed the presence of multiple stress- and hormone-responsive elements, indicating their potential regulatory functions in environmental adaptation (Figure 5). Expression profiling under various stress conditions and hormone treatments identified key OsC3H genes involved in ABA-, MeJA-, and drought-responsive pathways (Figure 7). Furthermore, haplotype analysis of OsC3H36 revealed two distinct allelic variants, with H1 contributing to increased grain width and 1000-grain weight (Figure 8), findings that were further validated using CSSL (Figure 9).

The functional diversification of OsC3H proteins observed in this study is consistent with findings in other plant species, such as *A. thaliana*, *Z. mays*, and *G. max*, where CCCH proteins have been implicated in RNA processing, stress adaptation, and developmental regulation [11,12]. Similar to *Arabidopsis* CCCH proteins, which regulate seed germination, flowering, and hormone signaling [14,15], several rice OsC3H genes exhibited tissue-specific and stress-induced expression, suggesting functional conservation across plant species. The identification of OsC3H40, OsC3H10, and OsC3H38 as key regulators of drought stress responses parallels previous findings that CCCH proteins can enhance abiotic stress tolerance via ABA- and JA-mediated pathways [46,47]. Notably, the upregulation of *OsC3H36* under multiple stress conditions and its association with grain weight align with previous reports demonstrating the role of CCCH proteins in modulating plant growth and productivity [48].

However, some discrepancies exist between our findings and previous studies. For instance, while *Arabidopsis* AtTZF1 functioned as both a positive and negative regulator of ABA and gibberellin (GA) signaling [19], no rice OsC3H genes exhibited similar dual regulatory behavior. This difference may stem from species-specific variations in CCCH protein function or differences in experimental conditions. Additionally, the unique C-X8-C-X5-C-X4-H motif identified in rice, absent in other species examined, suggests possible lineage-specific functional adaptations.

Our findings provide valuable insights into the regulatory mechanisms of CCCH zinc finger proteins in rice growth and stress responses. The identification of stress-responsive OsC3H genes offers potential targets for developing stress-resilient rice varieties through genetic engineering or molecular breeding. For example, *OsC3H40*, which exhibited significant upregulation under drought and ABA treatments, could serve as a candidate gene for improving drought tolerance in rice [46]. Furthermore, the discovery of haplotype variation in *OsC3H36* and its association with grain width and weight suggests that *OsC3H36* could be used in breeding programs to enhance rice yield. Given the importance of rice as a staple crop, these findings hold significant promise for ensuring food security under changing environmental conditions.

Despite the comprehensive nature of this study, several limitations should be acknowledged. First, while our expression analysis identified key OsC3H genes involved in stress responses, further functional validation through gene knockout or overexpression studies is needed to confirm their precise roles. Second, the regulatory mechanisms by which OsC3H genes mediate stress responses remain unclear, necessitating further investigation into their downstream target genes and interaction networks. Third, the impact of natural variation in OsC3H genes on rice agronomic traits warrants additional field trials under diverse environmental conditions to validate their potential utility in breeding programs. Future research should focus on elucidating the molecular mechanisms underlying OsC3H-mediated stress tolerance, particularly through transcriptomic and proteomic approaches. Additionally, genome editing techniques such as CRISPR/Cas9 could be employed to engineer desirable OsC3H alleles for improved rice stress resilience and yield optimization.

## 5. Conclusions

This study provides a comprehensive characterization of CCCH zinc finger proteins in rice, highlighting their evolutionary diversity, stress-responsive expression patterns, and potential roles in agronomic trait regulation. The identification of key OsC3H genes involved in drought tolerance and grain yield enhancement offers promising targets for rice improvement. Our findings contribute to a deeper understanding of CCCH zinc finger proteins in monocot species and pave the way for future functional studies and biotechnological applications in crop improvement.

## Figures and Tables

**Table 1:** Characteristics of the CCCH gene family in rice.

Gene Name	Gene ID	Isoelectric Point	Molecular Weight	Protein Length	Subcellular Location
*OsC3H1*	*LOC_Os01g07930*	8.69	29,860.95	279	nucleus
*OsC3H2*	*LOC_Os01g09620*	6.41	41,422.51	386	nucleus
*OsC3H3*	*LOC_Os01g14870*	8.88	17,888.01	167	nucleus
*OsC3H4*	*LOC_Os01g15300*	6.66	113,227.4	1007	nucleus
*OsC3H5*	*LOC_Os01g15350*	8.21	49,089.1	466	nucleus
*OsC3H6*	*LOC_Os01g15460*	7.50	49,834.79	476	nucleus
*OsC3H7*	*LOC_Os01g39100*	5.86	87,552.21	810	nucleus
*OsC3H8*	*LOC_Os01g42970*	6.90	50,358.15	461	nucleus
*OsC3H9*	*LOC_Os01g45730*	8.08	36,086.52	333	nucleus
*OsC3H10*	*LOC_Os01g53650*	6.06	24,982.17	225	nucleus
*OsC3H11*	*LOC_Os01g61830*	5.21	41,645.61	366	nucleus
*OsC3H12*	*LOC_Os01g68860*	8.89	47,852.24	439	nucleus
*OsC3H13*	*LOC_Os02g06584*	9.18	49,077.49	426	nucleus
*OsC3H14*	*LOC_Os02g10080*	9.58	31,153.44	300	nucleus
*OsC3H15*	*LOC_Os02g19804*	7.09	35,778.46	426	nucleus
*OsC3H16*	*LOC_Os02g35150*	8.85	78,052.14	678	nucleus
*OsC3H17*	*LOC_Os02g45480*	8.19	46,639.84	435	chloroplast
*OsC3H18*	*LOC_Os02g55000*	5.27	55,622.91	504	nucleus
*OsC3H19*	*LOC_Os02g58440*	5.29	71,469.18	647	nucleus
*OsC3H20*	*LOC_Os03g02160*	4.68	89,420.17	831	nucleus
*OsC3H21*	*LOC_Os03g05210*	6.47	46,317.29	427	chloroplast
*OsC3H22*	*LOC_Os03g18950*	5.88	48,549.22	457	nucleus
*OsC3H23*	*LOC_Os03g21140*	6.00	73,135.88	688	nucleus
*OsC3H24*	*LOC_Os03g21160*	6.07	71,950.88	677	nucleus
*OsC3H25*	*LOC_Os03g49170*	6.63	81,043.54	764	endoplasmic reticulum
*OsC3H26*	*LOC_Os03g61110*	9.25	36,258.57	312	nucleus
*OsC3H27*	*LOC_Os04g01480*	5.2	92,340.68	850	nucleus
*OsC3H28*	*LOC_Os04g02730*	6.25	75,572.46	685	nucleus
*OsC3H29*	*LOC_Os04g32340*	8.70	97,243.83	903	nucleus
*OsC3H30*	*LOC_Os04g35800*	9.61	38,697.91	346	nucleus
*OsC3H31*	*LOC_Os04g41060*	9.41	15,845.9	146	extracellular
*OsC3H32*	*LOC_Os04g48375*	8.38	34,286.86	315	cytosol
*OsC3H33*	*LOC_Os04g56750*	6.27	50,472.86	469	chloroplast
*OsC3H34*	*LOC_Os04g57010*	9.48	31,787.8	309	cytosol
*OsC3H35*	*LOC_Os04g57600*	5.33	80,159.97	711	nucleus
*OsC3H36*	*LOC_Os05g03760*	8.65	63,235.89	601	chloroplast
*OsC3H37*	*LOC_Os05g08400*	6.04	55,632.5	493	nucleus
*OsC3H38*	*LOC_Os05g10670*	9.02	49,684.7	464	chloroplast
*OsC3H39*	*LOC_Os05g41790*	9.25	76,541.3	703	nucleus
*OsC3H40*	*LOC_Os05g45020*	5.39	28,257.88	255	nucleus
*OsC3H41*	*LOC_Os05g48960*	9.25	34,692.17	304	nucleus
*OsC3H42*	*LOC_Os05g50080*	8.65	37,169.12	343	cytosol
*OsC3H43*	*LOC_Os06g07350*	8.12	54,009.89	482	chloroplast
*OsC3H44*	*LOC_Os06g21390*	7.96	41,673.18	368	nucleus
*OsC3H45*	*LOC_Os06g32720*	6.74	31,058.7	279	nucleus
*OsC3H46*	*LOC_Os06g32860*	6.01	80,907.75	711	nucleus
*OsC3H47*	*LOC_Os06g41384*	9.53	30,451.7	295	nucleus
*OsC3H48*	*LOC_Os06g41390*	6.85	64,563.78	600	nucleus
*OsC3H49*	*LOC_Os06g43120*	7.97	207,156.46	1890	nucleus
*OsC3H50*	*LOC_Os06g46400*	6.32	72,597.53	665	nucleus
*OsC3H51*	*LOC_Os06g46890*	9.24	42,478.83	372	nucleus
*OsC3H52*	*LOC_Os06g49080*	8.94	42,663.15	390	nucleus
*OsC3H53*	*LOC_Os07g04580*	6.72	40,200.85	388	nucleus
*OsC3H54*	*LOC_Os07g04650*	4.78	51,701.7	496	nucleus
*OsC3H55*	*LOC_Os07g18050*	6.72	54,334.99	486	cytosol
*OsC3H56*	*LOC_Os07g38090*	6.61	69,391.93	657	endoplasmic reticulum
*OsC3H57*	*LOC_Os07g39440*	9.07	49,917.89	438	nucleus
*OsC3H58*	*LOC_Os07g47240*	8.04	31,590.77	280	nucleus
*OsC3H59*	*LOC_Os07g48410*	6.15	72,494.47	682	nucleus
*OsC3H60*	*LOC_Os08g03310*	6.73	71,817.92	653	cytosol
*OsC3H61*	*LOC_Os08g04170*	8.76	105,583.58	958	nucleus
*OsC3H62*	*LOC_Os08g06330*	6.82	37,957.8	367	nucleus
*OsC3H63*	*LOC_Os08g38370*	5.69	68,736.17	605	nucleus
*OsC3H64*	*LOC_Os09g13530*	7.05	26,161.09	252	nucleus
*OsC3H65*	*LOC_Os09g19940*	8.11	57,894.38	527	chloroplast
*OsC3H66*	*LOC_Os09g31482*	8.92	34,087.52	290	nucleus
*OsC3H67*	*LOC_Os09g36090*	4.70	198,678.84	1835	nucleus
*OsC3H68*	*LOC_Os10g25220*	8.78	81,200.6	702	nucleus
*OsC3H69*	*LOC_Os11g28270*	8.77	48,006.1	444	nucleus
*OsC3H70*	*LOC_Os12g03554*	6.38	60,054.42	527	nucleus
*OsC3H71*	*LOC_Os12g18120*	6.14	57,306.11	529	nucleus
*OsC3H72*	*LOC_Os12g21700*	8.50	48,663.52	454	nucleus
*OsC3H73*	*LOC_Os12g33090*	6.04	64,725.96	619	plasma membrane

## Data Availability

No new data were created or analyzed in this study. Data sharing is not applicable to this article.

## Supplementary Materials

The following supporting information can be downloaded at: https://www.mdpi.com/article/10.3390/genes16040429/s1, Figure S1: Phylogenetic analysis of CCCH genes in rice and Arabidopsis; Figure S2: Synteny relationships between rice and dicots; Figure S3: Synteny relationships between rice and monocots; Table S1: Details of CCCH gene family in rice; Table S2: Duplicated gene pairs and Ka/Ks ratios of OsC3Hs in rice; Table S3: The number of cis-controlled elements; Table S4. Haplotype information.
