# Supplementary figures and images for: Identification and Expression Analysis of CCCH Zinc Finger Family Genes in Oryza sativa
Source: Genes (Basel). 2025 Apr 3;16(4):429. doi: 10.3390/genes16040429 (PMC12026475; doi:10.3390/genes16040429)

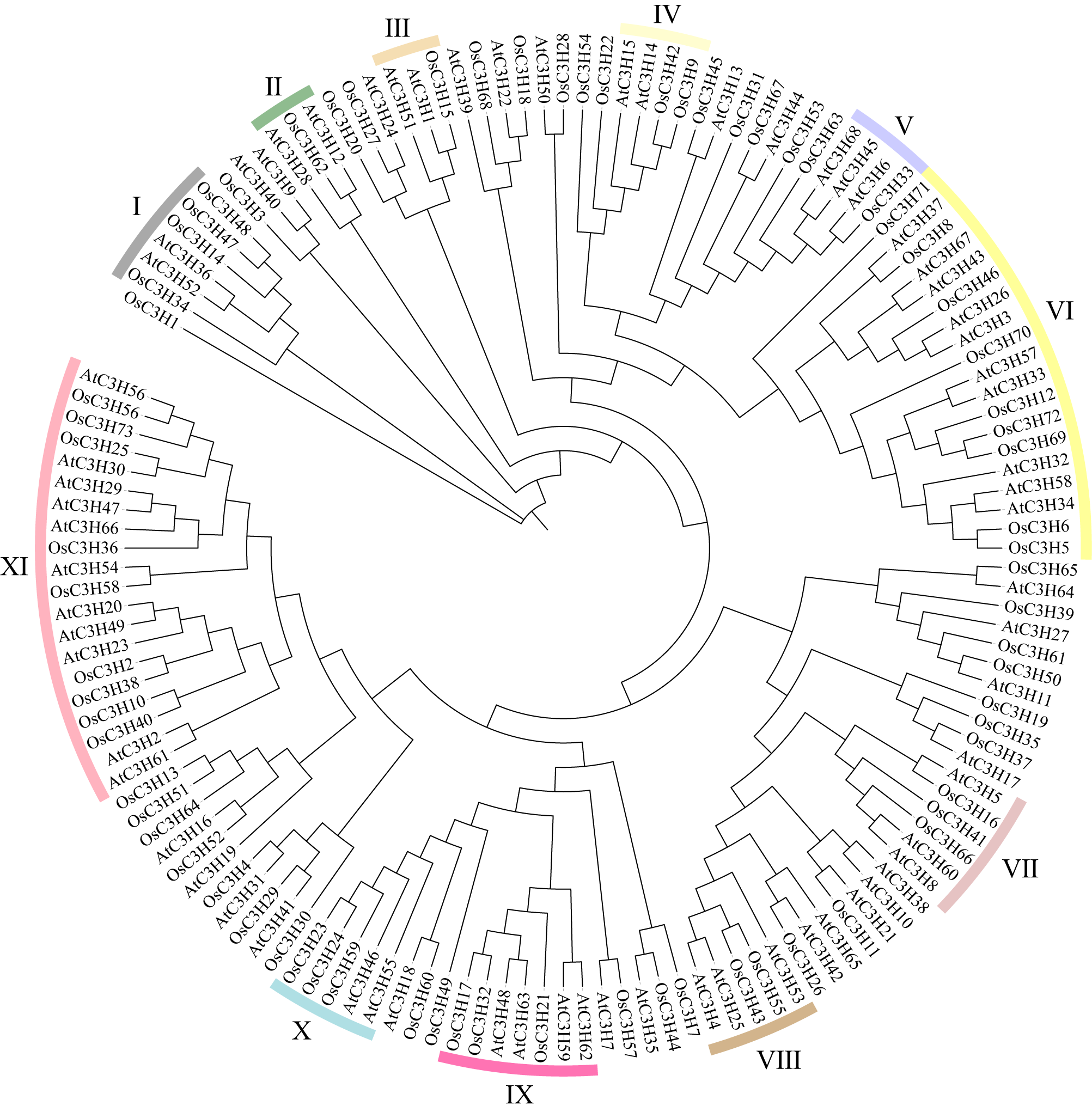

Supplement: Supplementary file 1 [file genes-16-00429-s001.zip › Figure S1.tif]

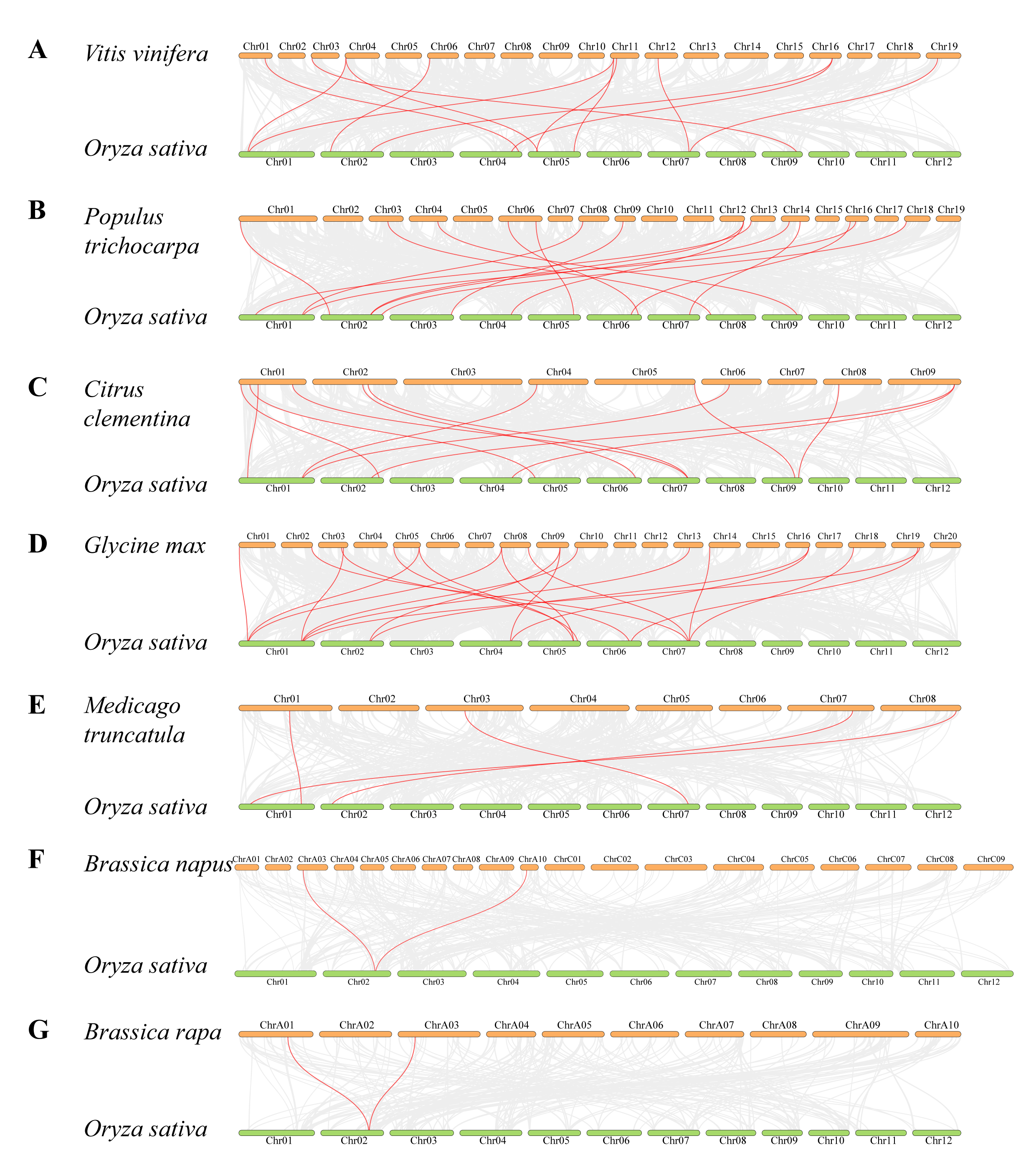

Supplement: Supplementary file 1 [file genes-16-00429-s001.zip › Figure S2.tif]

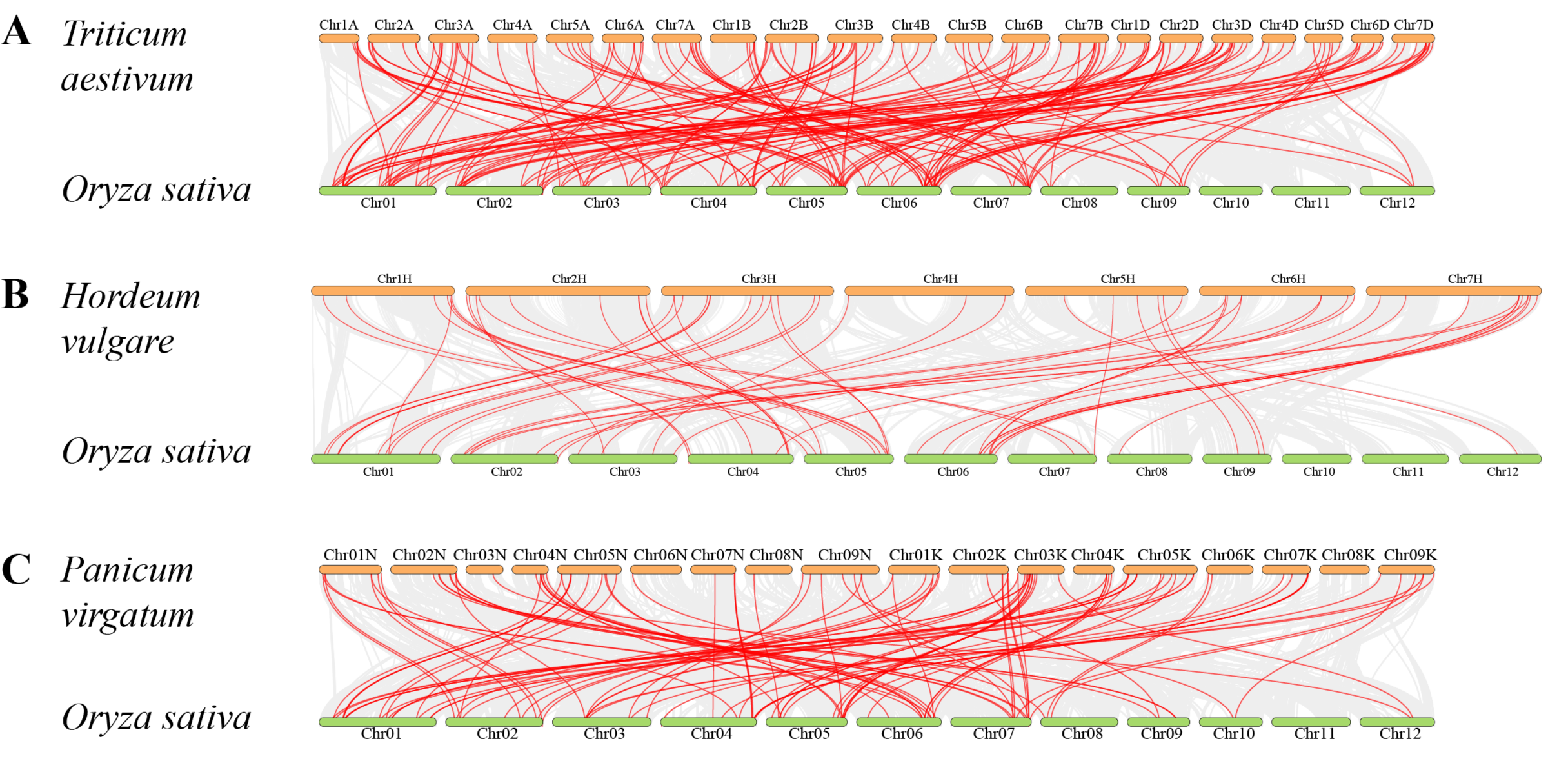

Supplement: Supplementary file 1 [file genes-16-00429-s001.zip › Figure S3.tif]
